# Effectiveness of acupuncture plus music therapy for post-stroke depression: Systematic review and meta-analysis

**DOI:** 10.1097/MD.0000000000039681

**Published:** 2024-09-13

**Authors:** Junyan Zhang, Yaowei Zhao, Hongyu Li, Yinyue Yang, Qiang Tang

**Affiliations:** a Heilongjiang University of Chinese Medicine, Harbin, China; b Rehabilitation Center, Second Affiliated Hospital of Heilongjiang University of Chinese Medicine, Harbin, China.

**Keywords:** acupuncture, meta-analysis, music therapy, post-stroke depression

## Abstract

**Background:**

Post-stroke depression (PSD) is a prevalent complication of stroke that adversely affects patient outcomes. The etiology of PSD is complex, and no universally effective treatment exists. Acupuncture, with its historical use, combined with music therapy, presents a novel approach for PSD treatment. This study aims to systematically evaluate the clinical efficacy of combining acupuncture with music therapy for PSD through a meta-analysis.

**Methods:**

We systematically searched both Chinese and English literature in PubMed, Embase, Web of Science, China National Knowledge Infrastructure, Wanfang, and the Chinese Science and Technology Periodical Database (VIP Database) for randomized controlled trials evaluating acupuncture combined with music therapy for PSD. Two independent evaluators conducted quality assessments and data extraction. Statistical analyses were performed using RevMan 5.4 and Stata 18.0 software.

**Results:**

This article contains 11 studies, involving a total of 698 patients. The results of the meta-analysis showed that, compared with the control group, the test group showed significant improvement on multiple outcome measures: HAMD score [mean difference (MD) = ‐3.18, 95% confidence interval (CI) (‐3.61, ‐2.76), *P* < .00001], Self-Rating Depression Scale score [MD = ‐5.12, 95% CI (‐6.61, ‐3.63), *P* < .00001], Pittsburgh sleep quality index score [MD = ‐2.40, 95% CI (‐2.96, ‐1.84), *P* < .00001], BI score [MD = 14.16, 95% CI (4.37, 23.94), *P* = .005] were all significantly lower, significantly higher effectiveness [risk ratio = 1.21, 95% CI (1.11, 1.33), *P* < .0001]. These differences were also statistically significant.

**Conclusion:**

The use of acupuncture combined with music therapy is effective in reducing depression in PSD patients.

## 1. Introduction

Post-stroke depression (PSD) occurs after a stroke and is a common psychiatric complication. A higher percentage of stroke survivors suffer from depression compared to normal individuals. Its main manifestations include symptoms such as persistent low mood, significant weight loss, insomnia or drowsiness, and untimely feelings of guilt.^[1]^ According to relevant statistics, the prevalence of post-stroke depression is as high as 40% to 50%, but misconceptions about the association between the presentation of symptoms and mood and the disease itself lead to post-stroke depression symptoms being masked, not taken seriously, and thus neglected for treatment.^[2]^ PSD severely affects the recovery of motor function, increases drug and alcohol use, and leads to a significant increase in mortality.^[3]^ Therefore, early detection and intervention in PSD are essential. Western medical treatment is usually based on oral antidepressants, such as selective serotonin reuptake inhibitors and tricyclic antidepressants.^[4]^ These medications are often associated with a higher risk of developing PSD. These drugs are often associated with strong side effects, which can seriously affect the extrapyramidal system, autonomic functions and may even lead to cerebral hemorrhage. Therefore, long-term use of these drugs is not recommended.

Chinese medicine is more effective than antidepressants, is safe, inexpensive, and has few side effects, which makes it popular in clinical practice.

Acupuncture therapy can promote the reorganization of the cerebral cortex network by stimulating specific acupoints, regulating neuroplasticity, relieving emotional stress, and improving the emotional state. It is targeted at treating the original disease while relieving the liver and resolving depression and it plays a role in treating the symptoms and the root cause of the disease. The sound waves of different frequencies transmitted to the brain can soothe one’s emotions and regulate the overall balance, as well as regulate sleep and suppress anxiety. As a “green” treatment, acupuncture supplemented by music therapy has been widely used in the treatment of post-stroke depression in recent years, and its efficacy has surpassed that of antidepressants, but there is less clinical literature on the subject. To promote the use of this method and strengthen the evidence of its effectiveness. Therefore, based on previous studies, we included clinical studies to conduct a meta-analysis of the effectiveness of acupuncture combined with music therapy in PSD, which provides a new way of thinking for the clinical treatment of PSD.

## 2. Methods

### 2.1. Date source and search strategy

A search was conducted in PubMed, Embase, Web of Science, China National Knowledge Infrastructure, Wanfang, and Chinese Science and Technology Periodical Database (VIP Database) databases for all relevant literature on the clinical randomized controlled trials of Acupuncture Combined with Music Therapy for the Treatment of Post-Stroke Depression, with the search period set from the time of database construction to April 18, 2024. A combination of subject and free word searches was used. The search formula was as follows:

Chinese search formula: (“脑卒中”OR“中风”OR“缺血性脑卒中”OR“脑出血”OR“脑梗死”OR“脑梗塞”OR“脑血管意外”)AND(“抑郁”OR“抑郁症”)AND(“针刺”OR“针灸”OR“针法”OR“头皮针”OR“温针灸”)AND(“音乐疗法”OR“五行音乐”OR“五音调神”);

English search formula: (“Stroke “OR “Cerebrovascular Accident “OR “Apoplexy, Cerebrovascular “OR “Vascular Accident, Brain “OR “Acute Stroke”)AND(“Depressive Disorder”)OR(“Disorder, Depressive”)OR(“Neurosis, Depressive”)OR(“Depressive Syndrome”)OR(“Melancholia”)AND(“Acupuncture Therapy “)OR(“Pharmacoacupuncture Treatment”)OR(“Pharmacoacupuncture Treatment”)AND(“Acupotomy”)AND(“Music Therapy”).

### 2.2. Literature inclusion criteria

#### 2.2.1. Study type

Clinical randomized controlled trials, whether blinded or unblinded, and no specific requirements for the language of the literature.

#### 2.2.2. Study subjects

Patients with a clear diagnosis of post-stroke depression according to different versions of the diagnostic criteria; With no restrictions on age, gender, or duration of illness, but with comparable baseline data; and no cognitive dysfunction or the ability to clearly express changes in their condition;

#### 2.2.3. Intervention study

The experimental group was treated with acupuncture combined with music therapy (acupuncture points and music type were not limited); the control group was treated with drugs or other conventional treatments; and the experimental group was not allowed to take antidepressants before treatment.

#### 2.2.4. Outcome indicators

Primary outcome indicators: overall effectiveness, Hamilton Depression Scale (HAMD), Self-Rating Depression Scale (SDS); secondary outcome indicators: Pittsburgh sleep quality index (PSQI), Barthel Index (BI).

#### 2.2.5. Exclusion criteria

(1) Review, conference abstract, case report, animal experiments, and case treatment literature.
(2) Literature published from the same experiment.
(3) The literature between the test group and the control group is not consistent and not comparable.
(4) Literature with incomplete data and impossible to merger data.

### 2.3. Data extraction and analysis

#### 2.3.1. Data extraction

Two researchers back-to-back independently screened the literature according to the inclusion and exclusion criteria. The data extraction form was designed using an Excel spreadsheet to perform data extraction in the included literature, and the extracted contents included: (1) basic information: the year of publication of the article, the country, the first author, and the baseline characteristics of the study subjects, etc. (2) Sample size of the experimental group and the control group, the interventions, and the time when the experiments were conducted, etc. (3) The data on the endpoint indexes in the various kinds of literature. (4) The content related to the assessment of the article’s quality. When exchanging and comparing the extracted contents with each other, in case of disagreement, the 2 researchers negotiated and resolved the issue, or referred to a third authoritative person to assist in the judgment, to ensure the accuracy and consistency of data extraction.

#### 2.3.2. Quality assessment

Evaluation was performed according to the Revman 5.4 built-in literature quality assessment tool under the Cochrane Collaboration, which mainly included: (1) randomized implementation; (2) allocation concealment; (3) double-blinding of implementers and participants; (4) implementation of blinding of outcome assessors; (5) completeness of outcome data; (6) presence of selective publication; and (7) other possible bias. Risk assessment results included high-risk, low-risk, and unclear.

#### 2.3.3. Statistical methods

Meta-analysis was performed using Revman 5.4 and Stata 18.0 software. Continuous variable effect indicators were expressed using mean difference (MD) values with confidence intervals (CI) set at 95%, and dichotomous variables were expressed using relative risk ratio values with CI set at 95%. Statistical significance was achieved at *P* < .05. The heterogeneity of the included literature was judged according to the size of the *I^2^* value, and when *P* ≥ .1 and *I^2^* < 50%, it was considered that the heterogeneity among the studies was small or nonexistent, and therefore a fixed-effects model was used. On the contrary, it was considered that there was significant heterogeneity among the included studies, so the random effects model was used, and the source of heterogeneity was further analyzed and found by sensitivity analysis or subgroup analysis. Publication bias detection was meaningful when ≥10 papers were included, and publication bias could be assessed by plotting a funnel plot or by combining quantitative data given by Egger test.

## 3. Results

### 3.1. Description of the selected studies

**Figure 1.**
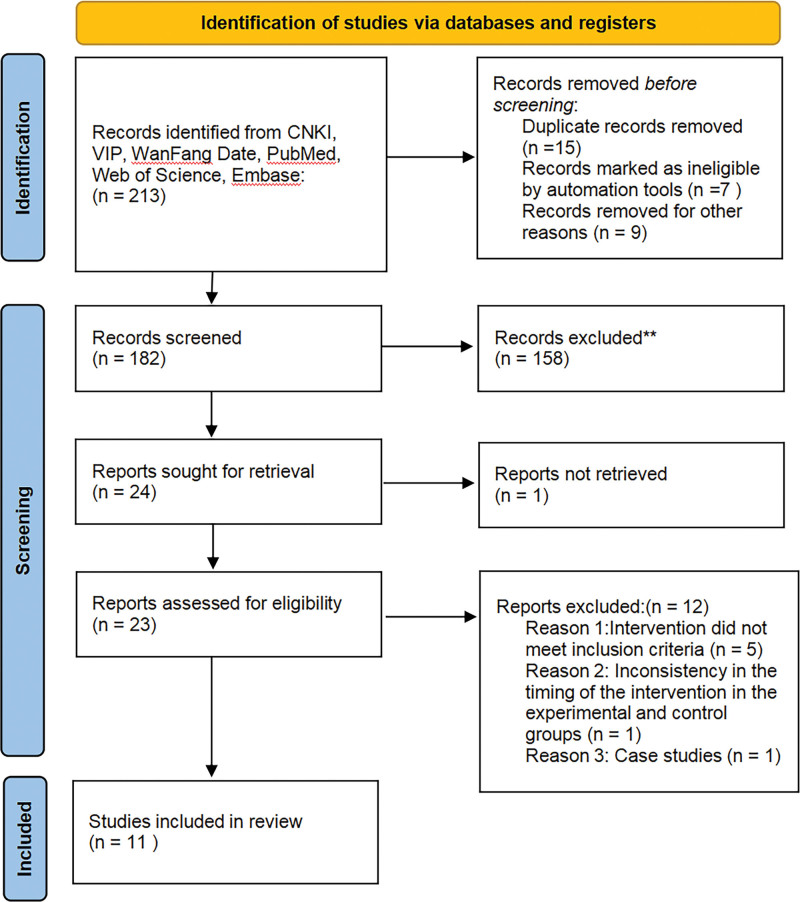
PRISMA flow chart. Databases that were searched included PubMed (n = 1), Web of science (n = 13), Embase (n = 98), CNKI (n = 53), Wanfang Data (n = 19), and VIP database (n = 28). CNKI = China national knowledge infrastructure; VIP = China Science and Technology Journal Database.

According to the search strategy in Chinese and English databases, a total of 213 articles were retrieved, and 182 articles remained after the literature check. After the initial screening, 158 documents were excluded, and 13 articles were excluded after rescreening. A total of 11 articles were finally included.^[5–15]^ The specific process is shown in Figure 1.

### 3.2. Characteristics of included studies

A total of 698 patients were included in the 11 included studies,^[5–15]^ with 350 in the trial group and 348 in the control group.

Interventions: the experimental group was acupuncture combined with music therapy; in the control group, 8 of the studies^[5,7,8,11–15]^ were acupuncture therapy; 2 studies^[6,10]^ were western drugs (escitalopram, fluoxetine hydrochloride) alone; and 1 study was repeated transcranial magnetic stimulation therapy.^[9]^ The duration of treatment ranged from 3 to 8 weeks.

Outcome indicators: 11 studies^[5–15]^ reported HAMD scores, 6 studies^[6,8,10,11,13,15]^ reported effectiveness rates, 2 studies^[7,11]^ reported BI scores, 4 studies^[7,9,11,12]^ reported PSQI scores, 3 studies^[12,13,15]^ reported SDS scores, as detailed in Table 1.

**Table 1:** Basic characteristics of the included studies.

Inclusion of studies	Sample size/case	Average age/year		Treatments	Intervention		Outcome indicator
	T/C	T	C		Test group	Control subjects	
Lin F 2017^[5]^	30/30	68.80 ± 11.529	72.93 ± 10.369	3 weeks	Five elements music + acupuncture	Treat by acupuncture	1
Liu Li 2021^[6]^	36/36	60.2 ± 3.5	60.1 ± 4.9	4 weeks	Five elements music + acupuncture	Escitalopram	1,5
Liu Yan 2021^[7]^	17/17	58.18 ± 5.25	57.76 ± 6.02	4 weeks	Five elements music + acupuncture	Treat by acupuncture	1,3,4
Zhang Pengyan 2022^[8]^	23/22	58.91 ± 5.93	60.09 ± 6.75	4 weeks	Five elements music + acupuncture	Treat by acupuncture	1,5
Wang Jian 2022^[9]^	36/35	59.75 ± 6.81	60.58 ± 6.95	6 weeks	Five elements music + acupuncture	Repetitive transcranial magnetic stimulation	1,3
Wang Chan 2022^[10]^	45/45	68.45 ± 4.44	68.04 ± 4.39	8 weeks	Five elements music + acupuncture	Fluoxetine hydrochloride	1,5
Wang Ning 2019^[11]^	30/30	49.53 ± 7.23	48.56 ± 7.82	4 weeks	Five elements music + acupuncture	Treat by acupuncture	1,3,4,5
Wang Min 2018^[12]^	40/40	44–68	45–66	4 weeks	Five elements music + acupuncture	Treat by acupuncture	1,2,3
Luo Jinfa 2020^[13]^	30/30	50.48 ± 7.15	51.55 ± 9.19	8 weeks	Five elements music + acupuncture	Treat by acupuncture	1,2,5
Zhao Deng 2022^[14]^	39/38	57.13 ± 6.59	57.07 ± 6.90	4 weeks	Five elements music + acupuncture	Treat by acupuncture	1
Huang Wei Ling 2021^[15]^	30/30	62.99 ± 6.49	63.51 ± 6.09	4 weeks	Five elements music + acupuncture	treat by acupuncture	1,2,5

The numbers correspond to the outcome indicators as follows: 1: Hamilton Depression Scale (HAMD); 2: depression self-assessment scale (SDS); 3: Pittsburgh sleep quality index (PSQI); 4: activities of daily living energy scale (BI); 6: effective rate.

C = control group, T = test group.

### 3.3. Estimation of quality

**Figure 2.**
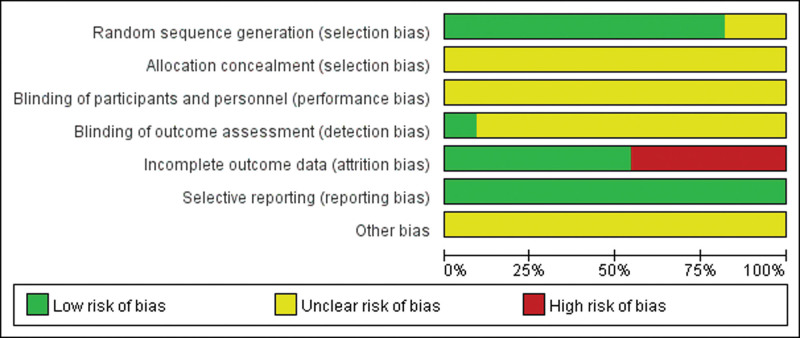
The risk of bias assessment diagram. *Note*: green: low risk of bias; yellow: unclear risk of bias; red: high risk of bias.

(1)Of the 11 studies included, 9 studies^[5–8,10,11,13–15]^ mentioned the use of the randomized table of numbers method of grouping. When assessed using the Cochrane Risk of Bias Assessment Tool in the randomized sequence produced one assessment of low risk, and 2 studies^[9,12]^ were not specifically described and therefore rated as unclear.
(2) One study^[14]^ had evaluators blinded at the time of the assessment of the outcome indicator, resulting in a low risk assessment.
(3) Five studies^[5,7–9,14]^ had cases dropout during treatment, so data completeness was assessed as high risk.
(4) In 11 studies,^[5–15]^ no selective reporting was present in any of the studies, so the assessment was low risk.
(5) Other elements (allocation concealment, other biases, etc) were unclear. The risk of bias assessment diagram is shown in Figure 2.

### 3.4. Meta-analysis of results

#### 3.4.1. The HAMD score

**Figure 3.**
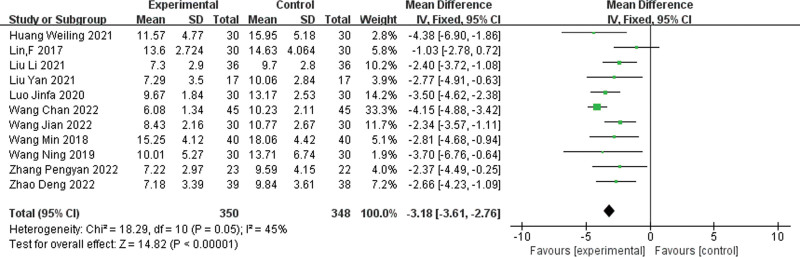
Forest plot for the Hamilton Depression Scale score. CI = confidence interval; df = degrees of freedom; IV = inverse variance methods; RR = rate ratio.

Eleven HAMD scores^[5–15]^ were reported in the literature, and the results of the heterogeneity test indicated that *P* = .05 and *I²* = 45%, which indicated that the heterogeneity among the studies was small. Therefore, a fixed-effects model was chosen to be used. The results of the meta-analysis showed that HAMD scores in the experimental group were significantly lower than those in the control group, and therefore the difference was statistically significant [MD = ‐3.18, 95% CI (‐3.61, ‐2.76), *P* < .00001]. Detailed results can be found in Figure 3.

#### 3.4.2. Clinical effective rate

**Figure 4.**
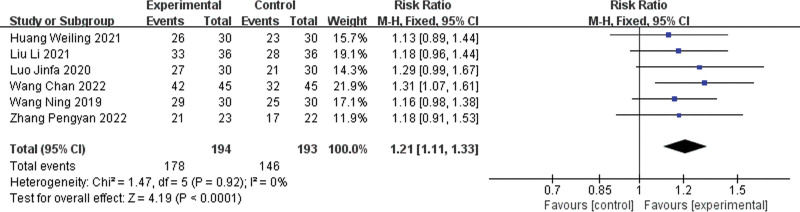
Forest plot for clinical effective rate.

Six clinical efficacy rates^[6,8,10,11,13,15]^ were reported in the literature, and the results of the heterogeneity test indicated that *P* = .92 and *I²* = 0%, suggesting that there was no heterogeneity among the studies, and therefore a fixed-effects model was chosen. The results of the meta-analysis showed that the efficacy rate of the experimental group was higher than that of the control group, and the difference was statistically significant [risk ratio = 1.21, 95% CI (1.11, 1.33), *P* < .0001]. Detailed results can be found in Figure 4.

#### 3.4.3. Depression self-rating scale score

**Figure 5.**
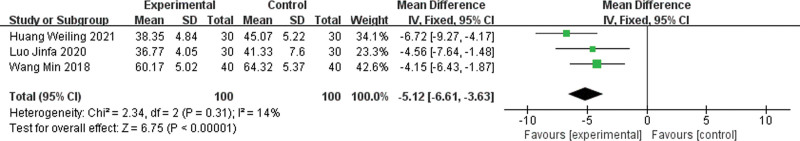
Forest plot for depression self-rating scale score.

Three SDS scores^[12,13,15]^ were reported in the literature, and the results of the heterogeneity test indicated that *P* = .31 and *I²* = 14%, suggesting that there was little heterogeneity among the studies, and therefore a fixed-effects model was chosen to be used. The results of the meta-analysis showed that SDS scores in the experimental group were significantly lower than those in the control group, and the difference was statistically significant [MD = ‐5.12, 95% *CI* (‐6.61, ‐3.63), *P* < .00001]. Detailed results can be found in Figure 5.

#### 3.4.4. PSQI score

**Figure 6.**
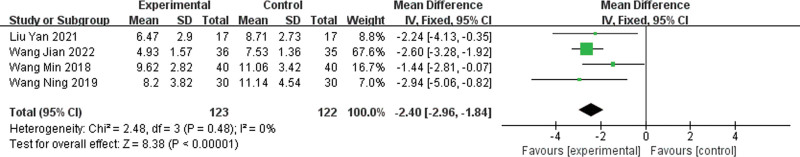
Forest plot for the Pittsburgh sleep quality index score.

Four PSQI scores^[7,9,11,12]^ were reported in the literature, and the results of the heterogeneity test indicated that *P* = .48 and *I*^2^ = 0%, which indicated no heterogeneity between studies, and therefore a fixed-effects model was chosen to be used. The meta-analysis showed that PSQI scores of the experimental group were lower than those of the control group. Therefore, the difference was statistically significant [MD = ‐2.40, 95% CI (‐2.96, ‐1.84), *P* < .00001]. Detailed results can be found in Figure 6.

#### 3.4.5. Activities of daily living energy scale (BI) score

**Figure 7.**
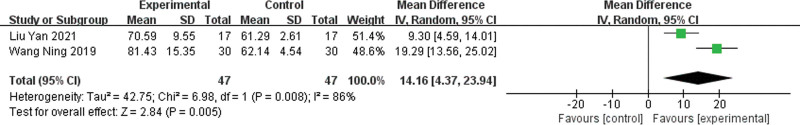
Forest plot for activities of daily living energy scale score.

Two PSQI scores^[7,11]^ were reported in one piece of literature, and the results of the heterogeneity test indicated that *P* = .008 and *I*^2^ = 86%, suggesting a high degree of heterogeneity among the studies, and therefore a random effects model was chosen to be used. However, due to the limited amount of literature, sensitivity analysis was not performed, and by reading the 2 studies, it was concluded that the heterogeneity might be related to the study design and the completeness of the data, which led to the bias of the results. The results of meta-analysis showed that the BI scores of the experimental group were significantly higher than those of the control group. Therefore, the difference was statistically significant [MD = 14.16, 95% CI (4.37,23.94), *P* = .005]. Detailed results can be found in Figure 7.

#### 3.4.6. Analysis of publication bias

**Figure 8.**
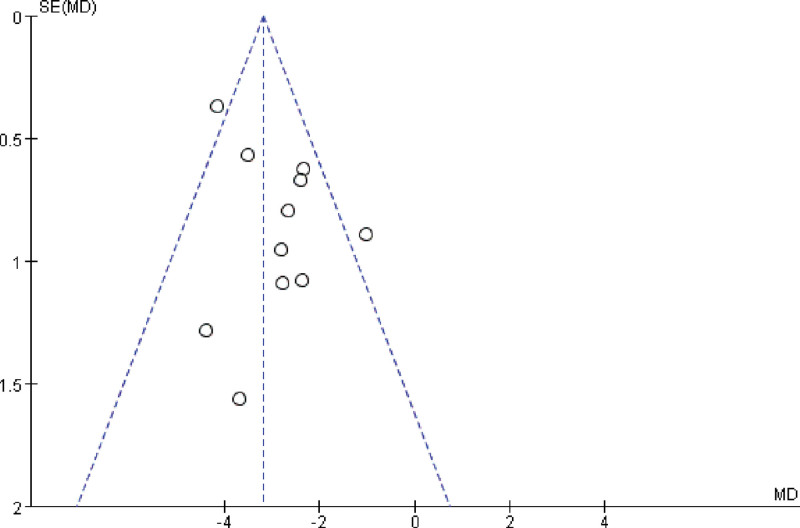
Funnel diagram for the Hamilton Depression Scale score.

A funnel plot of the HAMD scores from the 11 studies was created HAMD scores.^[5–15]^ By observation, the scatter points in the plot appeared to be generally symmetrical. However, some individual points were located outside the expected intervals, suggesting a potential for publication bias. Publication bias was further assessed quantitatively using statistical software (e.g., Stata 18.0), and the results of Egger test indicated that *P* = .130 > .05. Detailed results can be found in Figure 8.

## 4. Discussion

At present, the specific pathogenesis of post-stroke depression, as one of the most frequent complications of stroke, is still unclear, but the altered connections of the brain’s neural pathways and its neuronal function abnormalities and changes in cellular homeostasis are most closely related to its pathogenesis. A large number of mechanisms have been proposed to explain the pathogenesis of post-stroke depression, including neuroinflammation, abnormal activation of the hypothalamic–pituitary–adrenal axis, abnormal expression of vascular endothelial growth factor, reduction of brain-derived neurotrophic factor, and dysfunction of monoamine neurotransmitters (MNTs).^[16–18]^

Acupuncture has a long history in Chinese medicine treatment, based on the theory of acupuncture points of the human body, identification, and treatment through the meridians, leveling yin and yang.^[19]^ It is one of the preferred treatment modalities for depression, as the effect is rapid and significant with few side effects. “Suwen: Regulating the Meridian Theory” recorded: “Depending on its deficiency, press and lead to, prick and sharp, no blood, no diarrhea of its gas, in order to pass the meridian, the spirit is flat.” It can be seen that, although ancient times did not name “post-stroke depression.” The ancients already had knowledge of the treatment of such diseases with acupuncture, which was somewhat mature from holistic point of view, “depending on the deficiency of the network” on the basis of the original disease to be needled! The purpose of the treatment is to “level up the spirit and qi.”^[20]^ The aim of the treatment is to “level up the spirit and qi.” Back to modern research, it has been shown that acupuncture can regulate the balance of monoamine neurotransmitters, lower the level of inflammatory mediators, reduce the inflammatory response, regulate the hypothalamic–pituitary–adrenal axis, promote the regeneration of neural synapses, and increase the expression of brain-derived neurotrophic factors, thus playing an antidepressant role.^[4,21,22]^

“Music is also medicine.” Chinese medicine has been using music therapy to treat diseases for more than 2000 years. Music therapy is softer, and the healing effect can be regarded as the “and” method in the 8 methods, which can regulate Yin and Yang, reassure the spirit, and relax the emotions.^[23]^ Music therapy can be divided into different types. Music therapy is divided into different types, of which the 5 tones therapy is more widely used. Under the theory of the 5 elements, the 5-tone of “Pitch, Harmonic, Fundamental, Commercial, and Fifth” correspond to the 5 organs and the 5 emotions. For example, the horn tone is soothing and calming, which can ease the liver and relieve depression and regulate the flow of qi.^[24]^ The sound of the horn is soothing and calming, which can relieve the liver and depression and regulate the qi. Depending on the symptoms, corresponding music can be used for treatment. The excitability of the cerebral cortex increases with the incoming audio, the human body gradually enters a state of relaxation; and depression is gradually relieved under the effect of soothing audio. At the same time, music can reduce the aggravating factors of cerebral infarction (serum cortisol level) and inhibit cardiovascular stress.^[25]^ Listening to music has become an effective treatment for PSD.

Dong Jianping^[26]^ et al demonstrated that acupuncture therapy can effectively increase the content of 5-HT in the brain of PSD patients and restore hippocampal CA1 synaptic plasticity, thus acting as an antidepressant-like behavior, while the patients’ HAMD scores and SDS scores were also significantly reduced compared with those of the control group, and the overall effective rate was better than that of the control group. In animal experiments, Sun PY^[27]^ et al found that acupuncture increased the mRNA expression levels of brain-derived neurotrophic factor and tyrosine kinase receptor B (Trk B) and altered motor trajectory and sucrose preference, thereby suppressing depressive symptoms in mice. Sun Ruili^[28]^ et al chose different tunes according to the symptoms of PSD patients for 30 to 40 minutes each time and found that HAMD scores were significantly reduced and BI scores were elevated after 8 weeks, indicating that the quality of life of patients with alleviation of depressive symptoms was also improved. Yuan Bin^[29]^ found that combining acupuncture with the 5 elements of music therapy in mice, it was found that the effect of treatment with music therapy alone was relatively slow, while the combination with acupuncture therapy could significantly enhance the content of 5-HT and NE in the prefrontal cortex, hypothalamus, and hippocampus in rats with post-stroke depression, thus producing an antidepressant effect. In summary, I found that acupuncture and music therapy play an important role in the treatment of PSD, so I chose acupuncture combined with music therapy as the meta-analysis of PSD treatment modality, and the results showed that acupuncture combined with music therapy was consistent with the findings of the above study, and the effect of the combined use was better than the efficacy of either application alone.

In this study, 11 papers,^[5–15]^ 698 cases were summarized by meta-analysis, and the results of meta-analysis of clinical effectiveness showed that the use of acupuncture combined with music therapy was more effective than the use of acupuncture therapy alone, western medicine therapy alone, or repeated transcranial magnetic therapy. The results of meta-analysis of the scores of HAMD, SDS, PSQI, and BI scales showed that the use of acupuncture combined with music therapy could effectively reduce the depression level in PSD patients, improve the ability of daily life and sleep quality, and lay the foundation for the rehabilitation of good motor function in the later stage.

### 4.1. Limitations

However, the present meta-analysis still has some limitations: the study sample size is small, while the inclusion of a large number of foreign literatures are lacking. In the future, large samples and high-quality literature should be included to explore and analyze based on this direction. PSD pathogenic factors are related to family background, personality, and genetic factors, but when this analysis was conducted, the included literature did not classify patients for detailed description, the acupuncture method and music therapies typology were not classified for inclusion in the analysis, so there will be a slight difference in the efficacy of the treatment.

## 5. Conclusion

In conclusion, the use of acupuncture combined with music therapy can effectively lower the depression of PSD patients, and the therapy has a long history, high safety, low price, and is more suitable for patients with post-stroke depression who need long-term treatment to reduce the financial pressure of the family. There are certain advantages to the treatment, and it is worth further promoting its use.

## Author contributions

**Conceptualization:** Junyan Zhang, Yaowei Zhao, Qiang Tang.

**Data curation:** Junyan Zhang, Yaowei Zhao.

**Formal analysis:** Junyan Zhang, Hongyu Li, Qiang Tang.

**Funding acquisition:** Hongyu Li, Qiang Tang.

**Investigation:** Hongyu Li, Qiang Tang.

**Methodology:** Junyan Zhang, Qiang Tang.

**Resources:** Junyan Zhang.

**Software:** Junyan Zhang, Yaowei Zhao.

**Supervision:** Junyan Zhang.

**Validation:** Junyan Zhang, Yinyue Yang.

**Visualization:** Junyan Zhang.

**Writing – original draft:** Junyan Zhang.

**Writing – review & editing:** Junyan Zhang.

## References

[1] Hou J Zhang N Chen L. Advances in research on pathogenesis for patients with post-stroke depression. Chin J Stroke. 2010;5:852–856. [in Chinese].

[2] Hackett ML Köhler S O’Brien JT Mead GE. Neuropsychiatric outcomes of stroke. Lancet Neurol. 2014;13:525–34. 10.1016/S1474-4422(14)70016-X 24685278

[3] Kumar S. Sobering news about post-stroke depression. Lancet Psychiatry. 2017;4:2–3. 28012472 10.1016/S2215-0366(16)30410-2

[4] Villa RF Ferrari F Moretti A. Post-stroke depression: mechanisms and pharmacological treatment. Pharmacol Ther. 2018;184:131–44. 29128343 10.1016/j.pharmthera.2017.11.005

[5] Lin F Gu Y Wu Y Huang D He N. Effect of music therapy derived from the five elements in traditional Chinese medicine on post-stroke depression. J Tradit Chin Med. 2017;37:675–80. 32188229

[6] Liu L Ding Y Wang J. Clinical, gut flora, and serum serotonin responses to Wuyintiaoshen therapy in patients with mild-to-moderate post-stroke depression and liver Qi stagnation and spleen deficiency syndrome. Chin General Pract. 2021;24:3882–3887 [in Chinese].

[7] Liu Y Ba H Zhao D. Effect of music therapy based on scalp acupuncture on post-stroke depression: study with resting-state functional magnetic resonance imaging. Chin J Rehabil Theory Pract. 2021;27:282–289. [in Chinese].

[8] Zhang P Wang H Xie Q. Effect of “Wuyin Tiaoshen” (五音调神) method on post-stroke depression and related physical symptoms and serum 5-HT. Guiding J Tradit Chin Med Pharm. 2022;28:96–100 [in Chinese].

[9] Wang J Zhong H Zhu W. Application of five-tone divine method in patients with secondary insomnia and post-stroke depression. Chin Nurs Res. 2022;36:114–117 [in Chinese].

[10] Wang C Zu W Xu M. The regulatory effect of acupuncture method of inducing resuscitation plus five elements music therapy on monoamine neurotransmitters in patients with post-stroke depression. Clin J Chin Med. 2022;14:78–81 [in Chinese].

[11] Wang N Liu J Ding Y. Curative effect of “five-elements regulating mental activities” method on patients with post stroke depression. Rehabil Med. 2019;29:44–48 [in Chinese].

[12] Wang M Li L. Clinical observation of five elements music therapy combined with acupuncture for treating post-stroke depression. Shandong J Tradit Chin Med. 2018;37:906–908 + 919 [in Chinese].

[13] Luo J Lai Z Zhu Q. An analysis the treatment of 30 cases with post-stoke depression by Wuyin therapy combining with scalp acupuncture. Mod Chin Med. 2020;40:56–59 [in Chinese].

[14] Zhao D Liu Y Wang J. Effects of five pitches music combined with acupuncture on motor function and depression in patients with poststroke depression. Shandong J Tradit Chin Med. 2022;41:529–533 + 551 [in Chinese].

[15] Huang W-L Chen W Ng X-Y. Observation on the therapeutic effect of Zangshi Wuyin coordination acupuncture on post-stroke depression. World J Integr Tradit West Med. 2021;16:2334–2337 [in Chinese].

[16] Gong Q He Y. Depression, neuroimaging and connectomics: a selective overview. Biol Psychiatry. 2015;77:223–35. 25444171 10.1016/j.biopsych.2014.08.009

[17] Müller VI Cieslik EC Serbanescu I Laird AR Fox PT Eickhoff SB. Altered brain activity in unipolar depression revisited: meta-analyses of neuroimaging studies. JAMA Psychiatry. 2017;74:47–55. 27829086 10.1001/jamapsychiatry.2016.2783 PMC5293141

[18] Luo T Tian H Song H. Possible involvement of tissue plasminogen activator/brain-derived neurotrophic factor pathway in anti-depressant effects of electroacupuncture in chronic unpredictable mild stress-induced depression in rats. Front Psychiatry. 2020;11:63. 32153441 10.3389/fpsyt.2020.00063 PMC7044269

[19] Ban D. Development and modern clinical research of acupuncture manipulation. Inf Tradit Chin Med. 2013;30:126–128 [in Chinese].

[20] Zhang J Li F Geng L. Literature study on acupoint compatibility law of acupuncture in the treatment of post-stroke depression. World Chin Med. 2023;18:3353–3358 [in Chinese].

[21] Chen L Yao Z Qu S. Electroacupuncture improves synaptic plasticity by regulating the 5-HT1A receptor in the hippocampus of rats with chronic unpredictable mild stress. J Int Med Res. 2020;48:300060520918419. 32363965 10.1177/0300060520918419 PMC7221223

[22] Sun J Chen L Wang Z. Efficacy of acupuncture and moxibustion against depression and the mechanism: a review. World Chin Med. 2023;18:291–295 [in Chinese].

[23] Lin F-C. Mechanism of five notes of ancient China’s pentatonic scale in disease treatment based on meridian and collateral theory. J Beijing Univ Tradit Chin Med. 2019;42:465–468 [in Chinese].

[24] Lei B Dong F Xing J. An analysis of the idea of treating post-stroke depression syndrome from the theory of form and spirit by using five elements music. Glob Tradit Chin Med. 2020;13:56–59 [in Chinese].

[25] Barugh AJ Gray P Shenkin SD MacLullich AMJ Mead GE. Cortisol levels and the severity and outcomes of acute stroke: a systematic review. J Neurol. 2014;261:533–45. 24477489 10.1007/s00415-013-7231-5 PMC4928702

[26] Dong J Sun W Wang S. Clinical observation on head point-through-point electroacupuncture for treatment of poststroke depression. Chin Acupunct Moxibustion. 2007;27:241–44 [in Chinese]. 17585663

[27] Sun PY Chu HR Li N. [Effect of Tong du Tiao shen acupuncture on CREB/BDNF/Trk B signaling pathway of hippocampus in rats with post-stroke depression]. Chin Acupunct Moxibustion. 2022;42:907–13 [Chinese]. 10.13703/j.0255-2930.20220206-k0003 35938334

[28] Sun R Ren H Hao L. Application of five-element music therapy combined with positive psychological intervention in patients with depression following ischemic stroke: a research study. Chin Remedies Clin. 2020;20:2437–2439 [in Chinese].

[29] Yuan B. Effects of acupuncture combined with five-elements music on behavior and 5-HT, NE contents in different brain tissues of PSD rats. Nanjing Univ Chin Med. 2021 [in Chinese].

[30] Page MJ McKenzie JE Bossuyt PM. The PRISMA 2020 statement: an updated guideline for reporting systematic reviews. BMJ. 2021;372:n71. 33782057 10.1136/bmj.n71 PMC8005924

